# Developing an online cannabis store paradigm for Cannabis Regulatory Science

**DOI:** 10.1101/2025.10.20.25338387

**Published:** 2025-10-22

**Authors:** J.T. Borodovsky, S. Bairaboina, C.A. Struble, S.M. Preum, J.D. Sargent, J.A. Emond, A.J. Budney

**Affiliations:** a Center for Technology and Behavioral Health, Dartmouth Geisel School of Medicine, Lebanon, NH, USA; b Department of Biomedical Data Science, Dartmouth Geisel School of Medicine, Hanover, NH, USA; c Department of Psychological and Brain Sciences, Dartmouth College, Hanover, NH, USA; d Department of Psychology, University of Maine, Orono, ME, USA; e Department of Computer Science, Dartmouth College, Hanover, NH, USA; f Department of Pediatrics, Dartmouth Geisel School of Medicine, Hanover, NH, USA

**Keywords:** Cannabis Legalization, Online Dispensary, Point-of-Sale, Marketing, Advertising, Cannabis Regulation, Shopping Task

## Abstract

**Background:**

The legal cannabis industry is using online point-of-sale marketing, but few tools exist for evaluating how this marketing influences consumer behavior. To address this gap, we developed the Platform for Online Evaluation of Marijuana Marketing and Sales (POEMMS), a realistic, customizable online cannabis store. This study tested the feasibility, usability, and ecological validity of POEMMS.

**Methods:**

U.S. adults aged 18+ were recruited via social media advertisements. Participants engaged in a simulated shopping task using POEMMS and then completed a post-shopping survey. Participants were instructed to imagine having no cannabis and to buy products as they would in real life. Survey measures included typical real-world cannabis spending and 0–10 Likert ratings of the store’s usability and realism. Spearman correlations tested associations between in-store and self-reported spending.

**Results:**

N=678 completed the shopping task. Approximately 58% used cannabis daily. Median total spending was $100. Shopping task spending and self-reported spending for flower, open concentrates, cartridges, and edibles were strongly correlated (rs = 0.74, 0.74, 0.67, 0.62, respectively; all p<0.001). Participants rated the store as easy to navigate (Median=10) and product characteristics easy to find (Median=10). Perceived realism was also high across product names, types, potency, and descriptions (Median=8–10).

**Conclusions:**

POEMMS elicited behaviorally and perceptually valid responses in a large national sample of cannabis users. Findings demonstrate the platform’s utility for Cannabis Regulatory Science by allowing controlled evaluation of online marketing effects. Future applications of POEMMS could generate evidence to inform cannabis marketing policy and regulation.

## 1. INTRODUCTION

U.S. legal cannabis is a multi-billion-dollar industry that has been described as “a natural laboratory for marketing strategy research”(Olsen and Smith, 2019). In the wake of the COVID-19 pandemic, the industry has made a substantial shift to online sales and advertising (Israel, 2021; Jackson, 2020a, 2020b; Rup et al., 2020). However, regulatory frameworks have not kept pace with this transition, and thus, online cannabis retailers often operate with minimal oversight (Berg et al., 2023a). Consequently, cannabis store websites frequently employ aggressive point-of-sale (POS) marketing strategies that combine misleading pharmaceutical-style health claims and recreational lifestyle branding while omitting or downplaying adverse consequences and health warnings (Borodovsky et al., 2021; Borodovsky and Budney, 2018; Hoeper et al., 2022; Rhee and Jung, 2023). Given that cannabis consumers consider multiple product attributes beyond just price or potency (e.g., perceived quality, strain, packaging, recommendations) marketing strategies could shape purchase decisions and increase health risks among vulnerable subgroups such as young adults and those with mental health problems (Borodovsky and Budney, 2018; Donnan et al., 2022a, 2022b, 2024; Shi et al., 2019; Trangenstein et al., 2021; Zhu et al., 2021).

Despite rapid expansion of online cannabis retail, research on POS marketing within online stores remains limited, which leaves policymakers without the data needed to inform regulation. Much of the existing research on online cannabis marketing has focused on social media rather than the marketing used *within* online stores and/or use observational methods that do not allow for direct testing of marketing effects on purchase decisions (Duan et al., 2023; Marinello et al., 2024; Rup et al., 2020; Whitehill et al., 2020). Some studies have examined cannabis-related health claims, branding, and warning labels using survey-based experiments (Donnan et al., 2023; Goodman et al., 2019; Kowitt et al., 2022; Mutti-Packer et al., 2018), which limits ecological validity. Furthermore, studies that have investigated cannabis POS marketing have focused on brick-and-mortar stores (Berg et al., 2023b; Cao et al., 2020), rather than the store websites. Thus, there is a need for a research system that enables controlled experimental testing of online POS marketing strategies in a realistic cannabis shopping environment.

To address this gap, we developed the Platform for Online Evaluation of Marijuana Marketing and Sales (POEMMS)—a realistic, customizable online cannabis shopping platform. POEMMS allows researchers to control product attributes and display designs, implement various in-store advertising techniques (e.g., customer reviews and product recommendations, discounts, pop-up messaging, etc.), and track purchasing behavior in real time. This study presents pilot results on the feasibility of POEMMS for Cannabis Regulatory Science by evaluating the platform’s usability, realism, and validity.

## 2. METHODS

### 2.1. Platform Development and Structure

**Figure 1.**
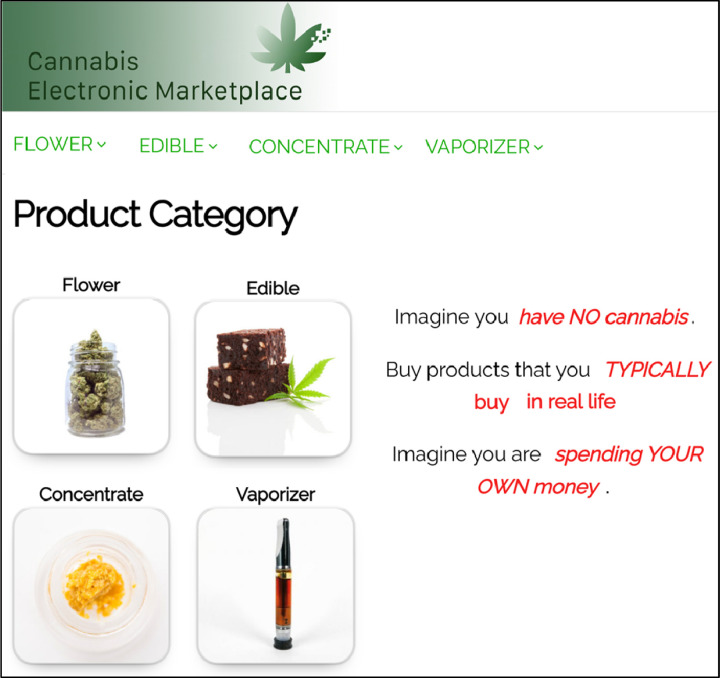
Screenshot of the homepage of the POEMMS online dispensary interface. POEMMS was built using WordPress^®^ and WooCommerce^®^ to mimic style and design elements of commercial cannabis retail websites. Participants selected from the four product categories (flower, edibles, open concentrates, and vaporizer cartridges). The standardized task instructions (red text), were displayed on all store pages.

**Figure 2.**
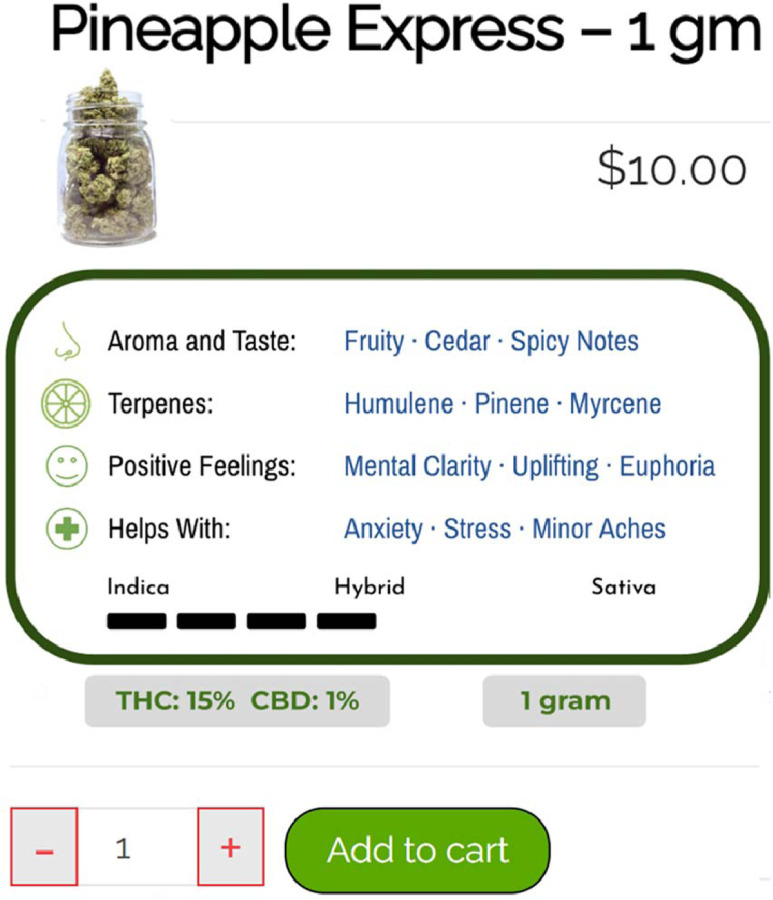
Screenshot of a POEMMS individual product page displaying product-specific information and purchase controls. Each product page included price, THC/CBD content, strain characteristics (e.g., aroma, terpenes, reported effects), indica-sativa profile, and an “add to cart” button. This content was standardized across products and designed to reflect common marketing elements used by commercial cannabis retailers.

POEMMS consists of a front-end online cannabis store and a back-end digital infrastructure for data collection and product stimuli manipulation. The platform was built using WordPress^®^ and WooCommerce^®^, which are the same systems used by many real online cannabis stores. The back-end infrastructure can track behaviors, including time spent browsing, page clicks, product views, and purchase decisions. For this initial study, the front-end store was populated with realistic cannabis product stimuli (e.g., pictures, names, potency, prices, and descriptions) based on peer-reviewed literature, expert consultation, and industry sources (e.g., Dutchie^®^, Wikileaf^®^, Leafly^®^). The initial version of the store was programmed to offer four popular product categories: flower/bud, open concentrates (e.g., wax, shatter), prefilled concentrate vaporizer cartridges, and edibles (Figure 1). Each product category contained six individual products that can vary on factors believed to affect purchase decisions, e.g., THC/CBD percentages, prices, and names. Participants can click on any of the six product variations to view a detailed individual product page displaying other factors that might affect purchase decisions (e.g., strain characteristics, expected effects) as well as an “add to cart” button (Figure 2).

### 2.2. Participant Recruitment and Eligibility

Participants were recruited (9/1/2023 to 10/31/2023) via Meta^®^ advertisements displaying a cannabis leaf and targeting U.S. adults aged 18+ with cannabis-related interests (e.g., Bob Marley)(Borodovsky et al., 2018). Participants were not required to have previously purchased cannabis online to participate. Advertisements directed individuals to the study landing page, where they provided consent and were redirected to the store. IP addresses were tracked to prevent duplicate responses. No participation compensation was provided. The study was approved by the Dartmouth Committee for the Protection of Human Subjects.

### 2.3. Shopping Task and Instructions

Participants were instructed to (1) imagine they had no cannabis, (2) buy products they typically purchase in real life, and (3) shop as if spending their own money.(Aston et al., 2021) These instructions were viewable on every webpage within the store (Figure 1). Shopping was self-paced. Participants also completed a post-shopping survey. The study instructions avoided revealing the primary research questions to mitigate demand characteristics.

### 2.4. Post-Shopping Survey

After shopping, participants completed a post-shopping survey that queried demographics, cannabis use patterns, and typical real-life spending amounts on cannabis flower, prefilled concentrate cartridges, open concentrates, and edibles. Specifically, participants were asked “When you buy cannabis in real life, about how much money do you typically spend on [flower/cartridges/concentrates/edibles]”. Additionally, to assess the usability of the store, participants used a Likert scale from 0 (Very Difficult) to 10 (Very Easy) to rate the difficulty of (1) navigating the store, (2) following the study instructions, and (3) finding relevant product characteristics (e.g., %THC, price). Participants also used a Likert scale from 0 (Very Fake) to 10 (Very Realistic) to rate the realism of the store product names, types, potencies, prices, and descriptions.

### 2.5. Analysis

We used descriptive statistics to summarize (1) demographics, (2) time spent shopping (3) shopping task purchase behaviors, (4) usability ratings, and (5) realism ratings. To assess the reliability of responses, we used Spearman correlations to compare purchase amounts observed in the shopping task to self-reported typical real-life purchase amounts. All analyses were conducted using Python 3.11.

## 3. RESULTS

### 3.1. Sample

N=852 participants consented, N=678 purchased at least one product, and N=579 answered at least one post-shopping survey question. Participant mean age was 36.6 years (SD: 12.9); 52.5% were female, and 80.0% were White. Nearly half (49.0%) reported an annual household income of ≥$50,000, and 37.7% had a bachelor’s degree or higher. More than half (57.9%) reported daily cannabis use in the past 30 days.

### 3.2. Shopping patterns

Participants shopped for a median of 2.58 minutes (IQR: 1.72–3.65 minutes) and viewed individual products for a median of 10 seconds (IQR: 5–16 seconds). Participants purchased a median of 2 (IQR: 2–4) individual products. Median total spending was $100 (IQR: $60-$194) and was highest for flower products (Median: $45, IQR: $15-$105) followed by prefilled cartridges (Median: $40, IQR: $0-$50), open concentrates (Median: $0, IQR: $0-$25), and edibles (Median: $0, IQR: $0-$20). These spending patterns are highly consistent with data from other published studies and industry reports (D’Amico et al., 2020; Gebru et al., 2023; Kepple et al., 2016; Kepple and Freisthler, 2018; McCann, 2024; Olson, 2015; Statista, 2020; Team Headset, 2020). Participants’ spending in the shopping task correlated strongly with their self-reported typical real life cannabis spending for flower (r_s_ = 0.74, p < 0.001), open concentrates (r_s_ = 0.74, p < 0.001), vaporizer cartridges (r_s_ = 0.67, p < 0.001) and edibles (r_s_ = 0.62, p < 0.001). Most participants (91.6%) agreed or strongly agreed that the products they purchased during the shopping task were similar to their usual real-world purchases.

### 3.3. Usability and Realism Ratings

Participants rated the store as easy to navigate (Median: 10, IQR: 8–10), the study instructions as easy to understand (Median: 10, IQR: 10–10), and product characteristics as easy to identify (Median: 10, IQR: 7–10). Realism ratings were high for product names (Median: 10, IQR: 8–10), product types (Median: 10, IQR: 8–10), potency (Median: 8, IQR: 6–10), and descriptions (Median: 9, IQR: 7–10). Realism ratings for prices were more uniformly distributed (Median: 6; IQR: 4–8.5).

## 4. DISCUSSION

This study examined the feasibility of the Platform for Online Evaluation of Marijuana Marketing and Sales (POEMMS) as a tool for studying cannabis purchasing behaviors in an online retail environment. Participants rated the store as highly realistic and easy to use, which suggests that the platform effectively replicates key aspects of the online cannabis shopping experience. Furthermore, POEMMS elicited shopping behaviors that were internally consistent with self-reported real-world cannabis spending, as reflected by strong correlations between in-store purchases and post-shopping survey responses. Observed spending patterns qualitatively mirrored purchase amounts reported in prior cannabis studies and industry data, suggesting that participants’ behavior in the simulated store resembled real-world consumer behavior. Overall, these results support the ecological validity of POEMMS and its potential as a tool for studying cannabis consumer decision-making.

As the online cannabis industry expands, understanding how point-of-sale marketing strategies influence consumer behavior will be increasingly important. POEMMS provides a structured environment for investigating this issue by balancing experimental control with ecological validity. Because the platform is highly customizable, researchers can systematically modify and test the impact of marketing-, pricing-, and policy-relevant variables, such as product descriptions, packaging, labeling, branding, customer reviews, bundled discounts, taxation strategies, warning labels, and health risk messaging. Findings from such studies could be used to understand how cannabis industry and regulatory practices shape consumer behavior and impact public health (Borodovsky et al., 2021).

One area of particular concern is the cannabis industry’s growing use of psychotherapeutic advertising claims (PACs)(Boatwright and Sperry, 2020; Duan et al., 2023; Hoeper et al., 2022; Lau et al., 2021; Luc et al., 2020). PACs contain mental health proxy terms (e.g., ‘happy,’ ‘relaxed,’ ‘uplifted’) and/or explicit treatment claims (e.g., ‘helps with depression,’ ‘relieves stress’) to imply that cannabis products are effective treatments for anxiety, depression, or other mental health conditions. This poses a serious risk to individuals seeking relief from symptoms of mental health conditions, as substantial evidence indicates that consuming Delta-9 tetrahydrocannabinol (primary intoxicating compound in cannabis) in large, acute doses or over prolonged periods can contribute to the onset of psychiatric conditions, worsen existing symptoms, and increase the risk of developing cannabis use disorder (Bahorik et al., 2018; Cuttler et al., 2018; Feingold et al., 2017; Hser et al., 2017; Kuhns et al., 2022; Moitra et al., 2021; National Academies of Sciences, Engineering, and Medicine et al., 2017; Patrick et al., 2016; Schoeler et al., 2018; van der Pol et al., 2013; Zvolensky et al., 2010). Future studies should examine how PACs shape purchasing behavior among individuals with mental health conditions and whether targeted mental health warning labels mitigate the effects of PACs.

This study has several limitations. First, participants were recruited through Meta^®^ advertising, and more than half of the sample consisted of daily or near-daily cannabis consumers. As a result, the findings may not fully generalize to less frequent or inexperienced consumers, who may respond differently to the stimuli used in the store (Borodovsky, 2022; Borodovsky et al., 2018). Second, participants made hypothetical purchases. Although this study and others (Aston et al., 2021, 2015; Aston and Cassidy, 2019) provide evidence supporting the validity of hypothetical cannabis purchasing, future studies could incorporate designs that compare real-world in-person purchases with hypothetical POEMMS purchases to further examine the validity of spending behaviors. Third, participants provided highly variable realism ratings for the product prices. Because the sample was drawn from across the US, this variability may reflect differences in state-specific cannabis pricing and tax structures. Future research may benefit from using state-specific pricing models and restricting recruitment to a single state to improve perceived pricing realism.

In sum, this pilot study supports the use of POEMMS to examine online cannabis purchasing behavior. The platform realistically replicated key aspects of the consumer shopping experience and produced data consistent with real-world purchasing patterns. The findings lay a foundation for future research on the impact of online point-of-sale marketing strategies on cannabis consumer decisions and for regulatory efforts to protect public health.
